# Dilemma of treating schizophrenia during pregnancy: a Case series and a review of literature

**DOI:** 10.1186/s12888-017-1475-z

**Published:** 2017-08-29

**Authors:** Andreea Teodorescu, Petru Ifteni, Marius Alexandru Moga, Victoria Burtea, Nicusor Bigiu

**Affiliations:** 1 Psychiatry and Neurology Hospital, 18th Mihai Eminescu Street, Brasov, Romania; 2 Transilvania University, Faculty of Medicine, 29th Eroilor Bvd, Brasov, Romania; 3 Clinical Hospital of Obstetrics and Gynecology, 36th George Baritiu Street, Brasov, Romania

**Keywords:** Schizophrenia, Pregnancy, Olanzapine, Antipsychotics

## Abstract

**Background:**

The choice of antipsychotic treatment during pregnancy remains controversial, mainly due to a lack of exposure and outcome data. Randomized clinical trials are practically impossible due to ethical reasons. Our reports describe three cases of closely monitored female patients with schizophrenia who were treated with olanzapine during pregnancy. The novelty of reports is that all patients were previously treated with olanzapine long acting injectable (LAI) for an average period of 3.8 years. During the LAI treatment period they were in remission and then refused to continue with LAI mainly due to treatment modality (injectable administration).

**Case presentation:**

The patients were relatively young, diagnosed with schizophrenia and were previously successfully treated with long acting injectable. The women were pregnant for the first time. In two cases, the patients had become pregnant during remission and they continued treatment with oral olanzapine. In the third case, olanzapine treatment was initiated during admission for a relapse.

**Conclusions:**

There are no controlled studies for the use of olanzapine therapy in pregnant women. More studies are needed to determine the effects of antipsychotics, including olanzapine, on pregnant women and the developing fetus. Schizophrenia relapse during pregnancy may expose the mother and the fetus to high risk if olanzapine is stopped. It is important to assess the risks and benefits of treating pregnant or breastfeeding women with antipsychotics, and weigh these against possible risks of anomalies and developmental problems to the fetus or child.

## Background

Women with schizophrenia may become pregnant, and motherhood is common in such women. The choice of antipsychotic treatment during pregnancy remains controversial, mainly due to a lack of exposure and outcome data [1]. Randomized clinical trials are practically impossible due to ethical reasons. Information regarding the safety of antipsychotic use during pregnancy is limited, creating a strong ethical dilemma [2]. The current literature on antipsychotic use during pregnancy and breastfeeding are mostly found in case reports and observational or retrospective studies [3].

Prescribing psychotropic medications during pregnancy is a complex issue involving (1) the risk of leaving a severe psychiatric illness untreated, (2) the attendant risk of complications to the mother, (3) the attendant risk of complications indirectly to the born baby and (4) the risk of teratogenic/embryo-lethal effects on the developing fetus [4, 5]. Second-generation antipsychotics have been used since the 1990’s. Olanzapine is placed among category C drugs by the US Food and Drug Administration *(*i.e., “Risk cannot be adequately ruled out. Animal and human studies have shown an adverse effect (i.e., teratogenic or embryo-lethal), but there are no adequate human studies”) and there is no unequivocal evidence of harm to the fetus [6].

None of the second generation antipsychotics (SGAs), including olanzapine, hold a licensed indication for treatment during pregnancy.

Our case reports describe three cases of closely monitored female patients with schizophrenia who were treated with olanzapine during pregnancy. We had obtained written informed consent for publication from all patients before submission. The study was approved by the Local Ethic Committee.

## Case presentation

### Case 1

This report is about a 31-year old female patient, married for 4 years, a university graduate, diagnosed with schizophrenia at the age of 21. She had previous psychiatric hospitalizations after stopping olanzapine treatment. The last psychiatric admission was in 2011. She agreed to follow a treatment with olanzapine LAI 300 mg once/month with an excellent outcome for almost 4 years. In February 2014, the patient refused LAI arguing that she had “had enough of injections”. She agreed to continue with oral olanzapine 10 mg/day. In May 2014, the patient requested counseling and psychiatric treatment. The psychiatric evaluation at that time revealed mild anxiety, absence of psychotic symptoms and a very good functioning level. The patient asked about the possibility of becoming pregnant under treatment and also about genetic inheritance of schizophrenia. The next evaluation was in September 2014 when she declared that she was 16 weeks pregnant, and that she had continued the treatment with olanzapine 10 mg/day and escitalopram 10 mg/day until the day before the consultation. Considering the current status and the patient’s wish to keep the pregnancy, the decision was made to continue olanzapine treatment. She has been monitored monthly throughout at a private obstetric setting.

Monthly psychiatric evaluations and telephone contacts with the patient and her husband have showed a good and sustained remission. She gave birth by Caesarean section to a normal healthy male child. The surgical intervention was made according to the local protocol for patients with psychotic disorder. The weight of the newborn was 3000 g, (Apgar score 10) with no signs of abnormalities. The BMI (body mass index) of the mother was 25,35 with normal vital signs. They were discharged after 5 days. Both she and her husband declared that the infant, now 2 years old, had had a normal development.

## Case 2

This report is about a 30 year old female diagnosed with schizophrenia at the age of 21 living with her mother and two brothers. The patient’s medical history revealed the bipolar disorder diagnosis of her mother and the trauma caused by her parents’ divorce at the age of 12. She had experienced multiple relapses due to lack of insight and treatment non-adherence. The treatment had included almost all antipsychotics, from haloperidol to clozapine. In 2011, after a long and severe psychotic episode, the patient and her family agreed to undergo treatment with olanzapine long acting injectable 300 mg twice/month. The patient’s evolution had been spectacular with no relapses during a 4-5 year period. She started to work in a small store and she had become socially involved. In December 2015, the patient informed the psychiatrist about her decision to continue treatment with oral olanzapine 10 mg/day instead of LAI. Her motivation was the long distance from home to the hospital and the 3 h period requested by the manufacturer for olanzapine LAI. She had been appointed monthly and evaluated by a board certificated psychiatrist and she had continued treatment with olanzapine 10 mg/day and zopiclon 7.5 mg/day for insomnia. The treatment had been administered and supervised during pregnancy by the family. She gave birth naturally at home (in a small village in the country-side) to a normal female baby (2800 g, Apgar score = 9). She was admitted in the same day to the Obstetrics Department for evaluation and mandatory vaccines. She was discharged one week later and she had continued treatment with olanzapine 10 mg/day. The baby was evaluated by a paediatrician at 3 months and then by a GP with normal developmental progress.

## Case 3

It is about a 33-year old female diagnosed with schizophrenia at the age of 20. The patient history revealed frequent episodes of violence especially towards her mother. In 2011, she started olanzapine LAI 300 mg twice/month. Her psychiatric status has been very much improved, with very good functioning compared with the pre-LAI period including a call-center job and a boyfriend. In September 2015, she decided to stop LAI and continued oral treatment for 2 more months then totally stopped medication. In February 2016, she was admitted in our emergency psychiatric unit after a violent conflict with her mother. She presented delusion of persecution, agitation and hostility. She was treated with olanzapine 10 mg BID and oral lorazepam 1 mg BID. After a routine investigation, we noticed that she was 25 weeks pregnant. Initially for safety reasons and then as a consequence of a legal order (she was accused of physical violence by her mother and a judge decided a long term hospitalization) she remained in a psychiatric acute setting during pregnancy. She had been monitored monthly by an obstetrician. In August 2016, she was transferred to the Obstetrics and Gynecology Department and she gave birth by Caesarian section to a 3300 g healthy male baby with normal glycaemia. The BMI of the mother was 28,37.

Six months later, the newborn was evaluated by a paediatrician and the developmental progress was appropriate. The patient was in remission and she had continued treatment with olanzapine 10 mg BID in hospital until the final legal decision.

## Discussion

In schizophrenia, during pregnancy, key risks include delayed recognition of pregnancy, less prenatal care, excessive smoking, failure to recognize and understand labor. There are patients with psychotic denial of pregnancy or delusional refusal of obstetric ultrasound examination. In these cases, the risks of unassisted delivery and failure to bond with the baby are higher. There are no routine treatment recommendations applied during pregnancy, and it is difficult to reach definitive conclusions regarding their safety for the developing child.

Our case reports describe three cases of female patients with schizophrenia who had continued oral treatment with olanzapine during pregnancy. It is very plausible that two of them were on treatment since week 1 of pregnancy (family statement and supervision) in a remission phase, and one on treatment from week 25, when she was admitted for relapse. The newborn babies were age appropriately developed. In the first case, it was an intended pregnancy, while in the other cases pregnancy occurred accidentally.

In our previous case report [7] we showed that olanzapine was well tolerated without any side-effects for the newborn when it was taken in the first trimester. More than that, the patient relapsed when they stopped medication after week 20 of pregnancy.

In a recent paper, from 610 pregnancies exposed to olanzapine there were 66% normal births, 9.8% premature births, 9.3% spontaneous abortions, 8% perinatal conditions and 4.4% congenital anomalies which did not appear to be an increased risk when compared with the general population [3].

FDA classifies medication risk during pregnancy into five categories to inform clinicians about the risks of fetus exposure. Categories include A (no risk in well-controlled human studies), B (no risk in animal studies), C (adverse effect on the fetus in animal studies, but no adequate studies in humans and potential benefits may warrant use of the drug in pregnant women despite potential risks), D (adverse effect on the fetus in animal studies and human investigational or marketing experience, but potential benefits may warrant use of the drug in pregnant women despite potential risks), and X (adverse effect on the fetus in animal studies and human investigational or marketing experience, and risks clearly outweigh potential benefits)^6^. Congenital defects like meningocele or ankyloblepharon [8], hip dysplasia [9], acheiria [10], and cardiovascular defect [11], have been reported in children previously exposed to olanzapine in utero but are quite similar with non-exposed cases.

The American Congress of Obstetricians and Gynecologists based on the available data of risks and benefits, recommends continuing pharmacotherapy during pregnancy as severe psychiatric episodes are generally thought to be caused by discontinuation of medication. Nevertheless, a psychotic mother can also act dangerously for herself and her fetus [12].

Olanzapine was found to be associated with low birth weight in a dose-dependent manner. Findings from 23 prospectively olanzapine-exposed pregnancies showed a 13% rate of spontaneous abortion, 5% stillbirth, 0% major malformations, and 5% prematurity, all within the range of normal control rates [13].

Another study of 18 pregnancies provided similar results, suggesting that olanzapine was relatively safe when used during pregnancy [14]. There was also one case report suggesting that the use of olanzapine during pregnancy was associated with neonatal hypoglycemia due to hyperinsulinemia [15] unconfirmed in our cases where the glucose level was normal, while insulin levels had not been taken (not mandatory in the local protocol).

The weights of all 3 newborns were 2800 g (girl), 3000 g and 3300 g (boys) considered as normal weight. Higher rates of low birth weight (LBW), defined as a birth weight of a liveborn infant of less than 2500 g regardless of gestational age and neonatal intensive care unit admission were reported in cases with olanzapine exposure during pregnancy compared with mothers who were treated with other atypical antipsychotics [16].

In another systematic review, authors evaluated 1090 first-trimester-exposed pregnancies with 38 malformations resulting in a malformation rate of 3.5% concluded that first-trimester exposure to olanzapine was not associated with an increased risk of congenital malformation [17].

In conclusion, the available data suggests that olanzapine can be used as first-line drug during first-trimester pregnancy. Other maternal factors relevant for the choice of an antipsychotic such as drug-specific adverse effect profile, previous response, weight gain, gestational diabetes, patient preference and drug availability must be taken into acount [18, 19]. The review of literature identified similar fetal outcomes in pregnancies exposed to olanzapine compared with outcomes reported in the general population [3].

These data may be useful to help guide clinicians and women decide to continue or discontinue olanzapine therapy during pregnancy, but should be considered within the limitations associated with limited data. Women should notify their clinicians if they become pregnant or intend to become pregnant while being treated with olanzapine or other psychotropics.

## Conclusions

There are no controlled studies for the use of olanzapine therapy in pregnant women. More studies are needed to determine the effects of antipsychotics, including olanzapine, on pregnant women and the developing fetus. Schizophrenia relapse during pregnancy may expose the mother and the fetus to high risk if olanzapine is stopped. The frequency of fetal outcomes in cases exposed to olanzapine did not differ from rates of outcomes reported in the general population. It is important to assess the risks and benefits of treating pregnant or breastfeeding women with antipsychotics, and weigh these against possible risks of anomalies and developmental problems to the fetus or child.

## Abbreviations

FDA: The Food and Drug Administration
FGAs: First Generation Antipsychotics
LAI: Long acting injectable
LBW: Low birth weight
SGAs: Second Generation Antipsychotics
TRS: Treatment resistant schizophrenia
